# Adverse effects of finerenone in patients with heart failure: a systematic review and meta-analysis

**DOI:** 10.3389/fcvm.2025.1601552

**Published:** 2025-05-27

**Authors:** Wanqian Yu, Fan Luo, Jingan Rao, Guangtao Lei, Qinghua Wu, Wen Shen, Pingping Yang, Ping Li

**Affiliations:** ^1^ Department of Cardiovascular Medicine, The Second Affiliated Hospital, Jiangxi Medical College, Nanchang University, Nanchang, Jiangxi, China; ^2^ Department of Gastroenterology, Jiangxi Provincial Hospital of Traditional Chinese Medicine, Nanchang, Jiangxi, China; ^3^ Department of Endocrinology and Metabolism, The Second Affiliated Hospital of Nanchang University, Nanchang, Jiangxi, China

**Keywords:** finerenone, heart failure, HFrEF, HFmrEF, HFpEF, adverse effects

## Abstract

**Background:**

Finerenone has been shown to improve outcomes in patients with heart failure (HF), encompassing those with reduced (HFrEF), mildly reduced (HFmrEF), or preserved ejection fraction (HFpEF). However, its clinical use is accompanied by notable adverse effects. This study aimed to evaluate the relative risks of adverse events associated with finerenone across HF phenotypes.

**Methods:**

A systematic search of PubMed, Embase, and Web of Science identified six randomized controlled trials involving 8,527 HF patients. The analysis considered hyperkalemia, hypotension, treatment-emergent adverse events (TEAEs), treatment-emergent serious adverse events (TESAEs), and treatment discontinuation due to adverse events.

**Results:**

Finerenone significantly increased the risk of hyperkalemia (RR = 2.07, 95% CI 1.77-2.44, *P* < 0.00001) and hypotension (RR = 1.49, 95% CI 1.31-1.68, *P* < 0.00001) compared to placebo, irrespective of HF phenotype. No significant differences were observed between finerenone and placebo in terms of TEAEs, TESAEs, or treatment discontinuation when analyzing the overall heart failure population. Compared to eplerenone, finerenone was associated with a lower risk of TEAEs (RR = 0.93, 95% CI: 0.89-0.98) and TESAEs (RR = 0.74, 95% CI: 0.66-0.84), with similar discontinuation rates. Additionally, one included study suggested that finerenone may have a lower risk of TEAEs (RR = 0.64, 95% CI 0.56-0.74), treatment discontinuation (RR = 0.37, 95% CI 0.25-0.54) and hyperkalemia (RR = 0.41, 95% CI 0.21-0.79) compared to spironolactone, with similar rates of hypotension (RR = 0.61, 95% CI 0.29-1.30) in HFrEF.

**Conclusion:**

Finerenone (10-25 mg) showed a similar safety profile to placebo, with no significant differences in TEAEs, TESAEs, or treatment discontinuation. Compared to eplerenone, finerenone was associated with fewer TEAEs and TESAEs, with comparable discontinuation rates. Moreover, in patients with HFrEF, finerenone may offer lower risks of TEAEs, treatment discontinuation, and hyperkalemia than spironolactone, with similar rates of hypotension.

## Introduction

Heart failure (HF) is a complex clinical syndrome with diverse etiologies, affecting approximately 56 million people worldwide. It imposes a substantial health burden, including diminished quality of life, frequent hospitalizations, increased healthcare costs, and high premature mortality rates (1). Despite notable advances in therapeutic interventions, such as SGLT-2 inhibitors and sacubitril/valsartan, these treatments are often accompanied by adverse effects, including symptomatic hypotension, angioedema, and urinary or genital infections (2, 3).

Mineralocorticoid receptor antagonists (MRAs) are well-established as a cornerstone therapy for reducing morbidity and mortality in patients with heart failure with reduced ejection fraction (HFrEF). Recent studies have demonstrated that finerenone, a novel non-steroidal MRA, significantly lowers the combined risk of heart failure worsening and cardiovascular death in patients with mildly reduced (HFmrEF) or preserved ejection fraction (HFpEF) compared to placebo (4).

However, while finerenone has shown efficacy in improving clinical outcomes, it is not without adverse effects. Discontinuation rates due to adverse reactions range from 3% to 13% (4, 5). Notably, randomized controlled trials (RCTs) involving patients with diabetes and kidney disease reported an increased risk of hyperkalemia (6, 7). In addition, the results of a secondary analysis of the FIDELIO-DKD (8) and FIGARO-DKD (9) studies showed that in patients with chronic kidney disease and type 2 diabetes with concomitant heart failure, although the risk of hyperkalemia caused by finerenone treatment was higher than that of placebo, it was not statistically significant in the FIGARO-DKD study (RR = 1.80, 95% CI: 0.93–3.45). Currently, there are limited safety data on the safety profile of finerenone across the full spectrum of HF phenotypes, including HFrEF, HFmrEF, and HFpEF. Thus, we conducted a systematic review to assess the relative risk of adverse reactions of finerenone in HF patients participating in randomized controlled trials, regardless of ejection fraction.

## Methods

### Protocol registration

We registered the protocol for this systematic review with PROSPERO (CRD42024611190) (https://www.crd.york.ac.uk/PROSPERO/).

### Data sources and search strategy

We conducted a comprehensive literature search in PubMed, Embase, and Web of Science databases up to November 6, 2024, using the following search terms: “heart failure” or “HF” or “ventricular dysfunction” and “finerenone” or “BAY 94–8862” and “randomized controlled trial.” To ensure inclusivity, we also reviewed the reference lists of retrieved articles to identify any additional relevant studies. The meta-analysis was performed and reported in accordance with the Preferred Reporting Items for Systematic Reviews and Meta-Analyses (PRISMA) guidelines (10).

### Selection criteria

Eligible studies had to meet the following inclusion criteria: (1) the enrolled participants had HF; (2) study design was a randomized controlled trial (RCT) of the treatment group (finerenone) and control group; and (3) trial reported adverse effects related to finerenone and provided outcome data [treatment-emergent adverse events (TEAEs), treatment-emergent serious adverse events (TESAEs), the discontinuation of treatment due to the adverse events, hypotension, and hyperkalaemia].

The exclusion criteria were as follows: (1) duplicated trials; (2) studies such as reviews, notes, conference abstracts, editorials, and so on; and (3) RCTs did not involve in finerenone. The details are shown in Table 1.

**Table 1:** Inclusion and exclusion criteria.

Category	Inclusion criteria	Exclusion criteria
Patient Population	HF, HFrEF defined as LVEF < 40% HFmrEF defined as LVEF (40%–49%) HFpEF defined as LVEF ≥ 50%	Not HF
Intervention/comparator	Finerenone and control group	Other drugs vs. control group
Outcome	TEAEs”, “TESAEs”, “hospitalization for HF”, “hypotension”, “hyperkalemia”, and “the discontinuation of treatment due to the adverse events”	No “TEAEs”, “TESAEs”, “hospitalization for HF”, “hypotension”, “hyperkalemia”, and “the discontinuation of treatment due to the adverse events” outcomes reported
Study design	RCT	Not-RCTs: reviews, meta-analysis, letter, conference abstracts, editorials, chapter
Language	English	Non-English language publications

HF, heart failure; HFrEF, heart failure with reduced ejection fraction; HFmrEF, heart failure with mildly reduced ejection fraction; HFpEF, heart failure with preserved ejection fraction; LVEF, left ventricular ejection fraction; RCT, randomized controlled trial. TEAEs, treatment-emergent adverse events; TESAEs, treatment-emergent serious adverse events.

### Data extraction and quality assessment

Wanqian Yu and Fan Luo independently extracted data and assessed the quality of the studies from the electronic database. The relevant data we extracted included the following: the baseline characteristics of the trials, interventions, comparisons, sample size, medication used, and follow-up duration. The reported outcomes included treatment-emergent adverse events (TEAEs), such as cardiac disorders (e.g., angina pectoris, sinus tachycardia), gastrointestinal disorders (e.g., constipation, flatulence, nausea), and abnormalities identified through investigations (e.g., elevated blood creatine phosphokinase levels, increased blood glucose levels). Additional TEAEs encompassed metabolism and nutrition disorders (e.g., diabetes mellitus, hyperkalemia), nervous system disorders (e.g., dizziness, headache), renal disorders, vascular disorders, and hypotension, among others.Treatment-emergent serious adverse events (TESAEs) were defined as events that: (1) resulted in death, (2) were life-threatening, (3) required inpatient hospitalization or prolonged an existing hospitalization, (4) caused persistent or significant disability/incapacity, (5) involved congenital abnormalities or birth defects, or (6) were deemed serious or medically significant by the investigator. Treatment discontinuation due to adverse events, including hypotension and hyperkalemia, was also documented. Disagreements were resolved through discussion with a third author (QH-W). In accordance with the study registration or protocol for each included study, as well as additional relevant information from ClinicalTrials.gov, three reviewers (WQ-Y, F-L, and QH-W) evaluated the randomization process, intended interventions, missing outcome data, outcome measurements, and the selection of reported results.

### Risk of bias assessment

The methodological quality of the four included RCTs was assessed by using the Cochrane Collaboration Risk of Bias Tool (Review Manager 5.4.1), which included the following sections: selection, performance, detection, attrition, reporting, and other biases. The results are shown in Supplementary Figure S1.

### Statistical analysis

Data analysis was conducted using Review Manager Version 5.4.1. Adverse effects outcomes were assessed as dichotomous variables and compared between the finerenone and control groups (placebo or eplerenone). Pooled odds ratios (ORs) or risk ratios (RRs) with 95% confidence intervals (CIs) were calculated by the Mantel– Haenszel random-effects model were used as summary statistics for the incidence of adverse outcomes in patients with HF who received ﬁnerenone vs. control.

Heterogeneity was evaluated using the I² statistic and Cochran's *χ*² test. An *I*² value <50% and *P* > 0.10 indicated low heterogeneity, and a fixed-effects model was applied. For *I*² > 50% or *P* < 0.10, significant heterogeneity was identified, warranting further analysis. Sensitivity analysis was conducted for *I*² > 50% by sequentially excluding individual studies and reanalyzing the remaining datasets.

Publication bias was assessed using funnel plots. Symmetry in the funnel plot suggested no significant publication bias. All *P*-values were two-tailed, with statistical significance set at 0.05 and CIs reported at the 95% level.

### Sensitivity analyses

Sensitivity analyses were conducted for the primary outcome by (1) using ﬁxed-effect models (The fixed effects model assumes that the effect size is the same for all included studies, with only random errors. This assumption allows analysis results to be concentrated on a common effect estimate, reducing the impact of heterogeneity between different studies.); and (2) sequentially deleting each study and reanalysing the datasets of all remaining studies (By systematically removing individual studies and reanalyzing the data, one can assess whether the results are significantly influenced by the inclusion of a specific study. This approach helps identify potential biases or instabilities. For instance, if the results fluctuate considerably after the removal of certain studies, it suggests that these studies may be critical factors influencing the overall conclusion. This process further clarifies which variables have the most substantial impact on the findings).

## Results

### Description of the study selection process and study characteristics

**Figure 1:**
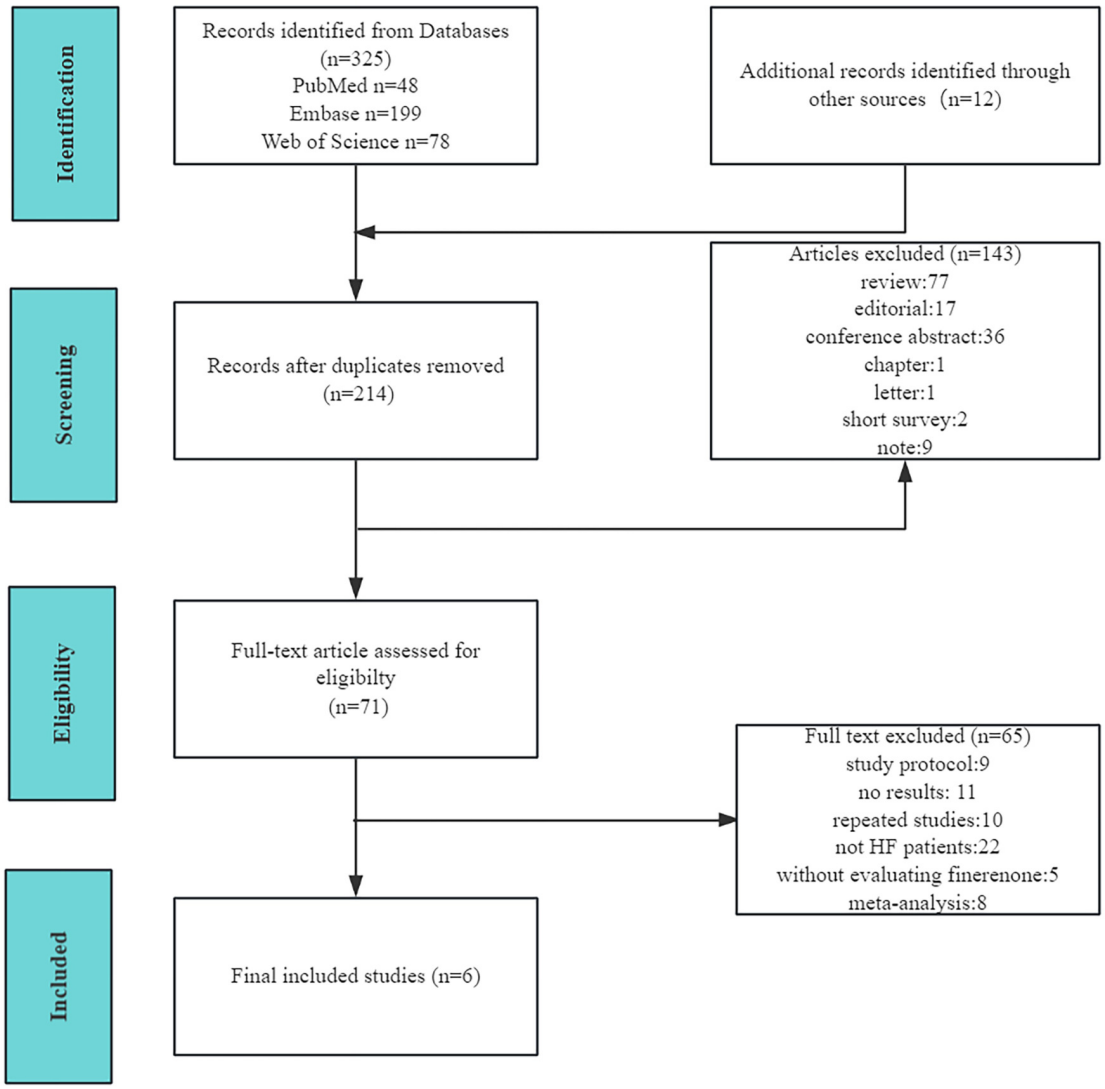
PRISMA diagram of the study selection process for the meta-analysis.

The detailed study selection process is illustrated in Figure 1. Ultimately, six double-blind randomized controlled trials (RCTs) involving a total of 8,527 patients were included in our analysis. Of these, three RCTs focused on patients with HFrEF, one included patients with HFmrEF or HFpEF, and two enrolled patients with HF while excluding those with symptomatic HFrEF.

The baseline characteristics of the included studies, such as follow-up duration, HF classification, and demographic data, are summarized in Table 2. Notably, compared to the HFrEF trials, the HFmrEF and HFpEF trials reported a higher prevalence of diabetes but a lower proportion of male participants. Additionally, we performed a baseline comparison across the included studies to assess potential differences in the populations at baseline (Supplementary Tables S1A,B).

**Table 2:** Baseline characteristics of RCTs.

Study	Group	Follow-up duration	HF setting	Age, year	Male (%)	DM (%)	Dose
ARTS Pitt et al. 2013 (5)	Finerenone (*N* = 264) *n* = 66	29 ± 2 day	HFrEF	71.2 (46–85)	52 (78.8)	20 (30.3)	2.5 mg q.d
	*n* = 67			72.0 (51–86)	55 (82.1)	21 (31.3)	5.0 mg q.d
	*n* = 67			72.5 (52–89)	59 (88.1)	25 (37.3)	10.0 mg q.d
	*n* = 64			71.9 (44–88)	46 (71.9)	18 (28.1)	5.0 mg b.i.d
	Spironolactone (*n* = 63)			72.8 (40–89)	50 (79.4)	24 (38.1)	25–50 mg q.d
	Placebo (*n* = 65)			72.4 (51–85)	50 (76.9)	26 (40.0)	NA
ARTS-HF Filippatos et al. 2016 (12)	Finerenone (*N* = 834) *n* = 172	90 days	HFrEF	72.5 ± 9.5	135 (78.5)	39 (22.7)	2.0–5.0 mg q.d
	*n* = 163			71.8 ± 10.6	126 (77.3)	36 (22.1)	5.0–10.0 mg q.d
	*n* = 167			69.3 ± 9.8	124 (74.3)	49 (29.3)	7.5–15.0 mg q.d
	*n* = 169			71.3 ± 10.2	128 (75.7)	48 (28.4)	10.0–20.0 mg q.d
	*n* = 163			69.2 ± 10.2	132 (81.0)	53 (32.5)	15.0–20.0 mg q.d
	Eplerenone (*n* = 221)			72.4 ± 9.9	170 (76.9)	55 (24.9)	25–50 mg q.d
ARTS-HF Japan Sato et al. 2016 (11)	Finerenone (*N* = 59) *n* = 13	90 days	HFrEF	73.2	11 (84.6)	2 (15.4)	2.0–5.0 mg q.d
	*n* = 13			71.2	9 (69.2)	2 (15.4)	5.0–10.0 mg q.d
	*n* = 11			78.2	6 (54.5)	3 (27.3)	7.5–15.0 mg q.d
	*n* = 11			65.9	8 (72.7)	1 (9.1)	10.0–20.0 mg q.d
	*n* = 11			73.5	7 (63.6)	1 (9.1)	15.0–20.0 mg q.d
	Eplerenone (*n* = 13)			76.5	12 (92.3)	3 (23.1)	25–50 mg q.d
FIDELIO-DKD Filippatos et al. 2022 (8)	Finerenone (*n* = 195)	2.6 years	HF^a^	66.4	120 (61.5)		10 mg or 20 mg q.d
	Placebo (*n* = 241)			66.2	160 (66.4)		NA
FIGARO-DKD Filippatos et al. 2022 (9)	Finerenone (*n* = 290)	3.4 years	HF^a^	64.9	182 (62.8)		10 mg or 20 mg q.d
	Placebo (*n* = 281)			66.3	168 (59.8)		NA
FINEARTS-HF Solomon et al. 2024 (4)	Finerenone (*n* = 3,003) Placebo (*n* = 2,998)	32 months	HFmrEF or HFpEF	71.9 ± 9.6 72.0 ± 9.7	1,648 (54.9 1,621 (54.1)	1,217 (40.5) 1,222 (40.8)	25 mg or 40 mg q.d NA

HF, heart failure; HFrEF, heart failure with reduced ejection fraction; HFmrEF, heart failure with mildly reduced ejection fraction; HFpEF, heart failure with preserved ejection fraction; DM, diabetes mellitus; q.d, one a day; b.i.d, twice a day; NA, not applicable. ^a^Exclusion of patients with symptomatic HFrEF.

### Adverse events of interest

#### TEAEs in patients with HF

**Figure 2:**
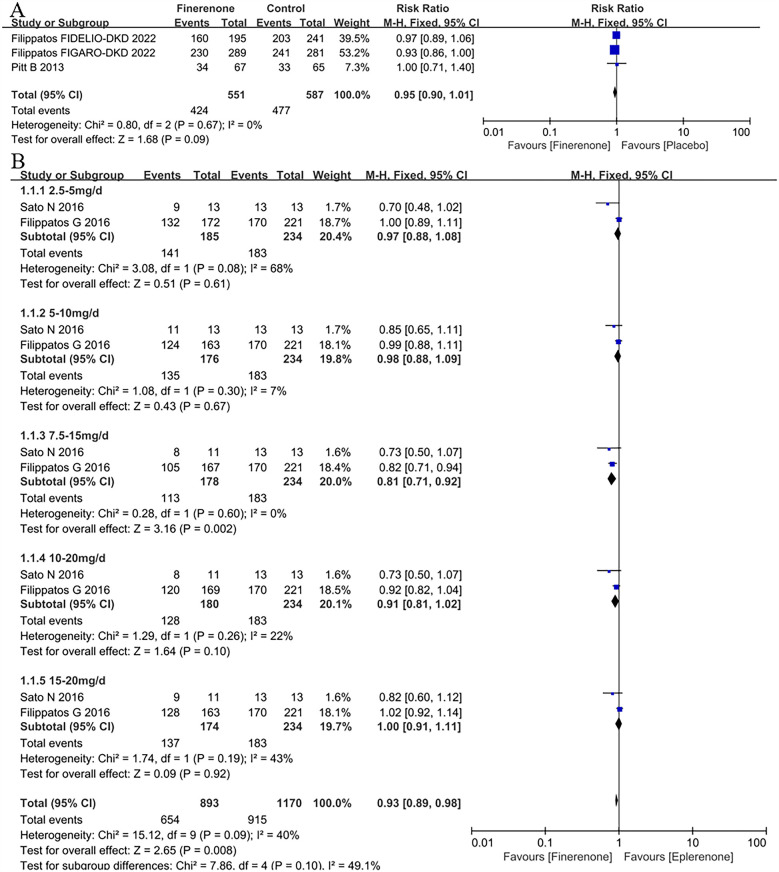
Forest plot of the outcomes of TEAEs. **(A)** TEAEs in patients with HF. **(B)** TEAEs in patients with HFrEF with different dose finerenone. TEAEs, treatment-emergent adverse events; HF, heart failure; HFrEF, heart failure with reduced ejection fraction; HFmrEF, heart failure with mildly reduced ejection fraction; HFpEF, heart failure with preserved ejection fraction.

A total of 3 articles (5, 8, 9) were included the analysis of treatment-emergent adverse events (TEAEs) in patient with HF. The overall risk of TEAEs across both treatment groups (finerenone and placebo) was 79.2%, with a slightly higher risk observed in the placebo group (81.3%) compared to the finerenone group (77.0%) (Figure 2A). However, the difference between the two groups was not statistically significant (RR = 0.95, 95% CI = 0.90–1.01, *P* = 0.09).

#### TEAEs in patients with HFrEF with different dose finerenone

Two RCTs (11, 12) were included in the analysis of TEAEs associated with different doses of finerenone. The results indicated that finerenone posed a lower risk of TEAEs compared to eplerenone (RR = 0.93, 95% CI: 0.89–0.98, *P* = 0.008). Subgroup analysis further revealed that the 7.5–15 mg dose group had a significantly lower risk of TEAEs than eplerenone (RR = 0.81, 95% CI: 0.71–0.92, *P* = 0.002), as shown in Figure 2B.

#### TESAEs in patients with HF

**Figure 3:**
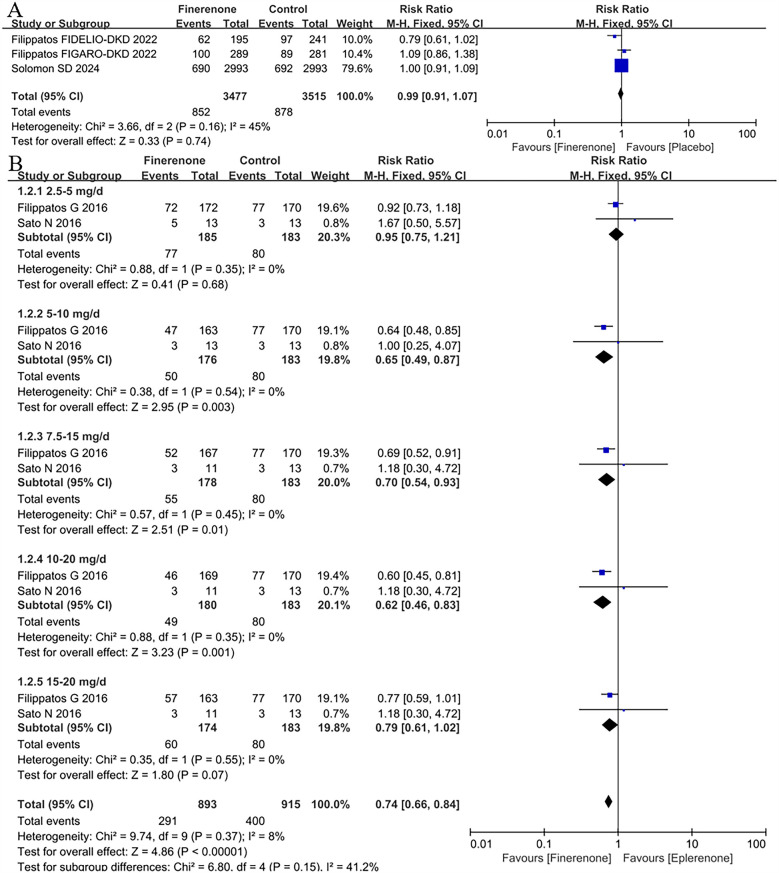
Forest plot of the outcomes of TESAEs. **(A)** TESAEs in patients with HF. **(B)** TESAEs in patients with HFrEF with different dose finerenone. TESAEs, treatment-emergent serious adverse events; HF, heart failure; HFrEF, heart failure with reduced ejection fraction; HFmrEF, heart failure with mildly reduced ejection fraction; HFpEF, heart failure with preserved ejection fraction.

3 RCTs (8, 9, 13) were included the analysis of treatment-emergent serious adverse events (TESAEs). In the overall population, the risk of TESAEs was 24.7%, with the lower risk among patients treated with finerenone (24.5%) and the higher risk in those with placebo (25.0%) (Figure 3A), whereas there was no difference between finerenone and placebo (RR = 0.99, 95% CI 0.91–1.07, *P* = 0.74).

#### TESAEs in patients with HFrEF with different dose finerenone

2 RCTs (11, 12) were included the analysis of TESAEs to compare different dose finerenone with eplerenone. The results showed that finerenone had a lower risk of TESAEs than eplerenone (RR = 0.74,95% CI:0.66–0.84, *P* < 0.00001); subgroup analysis showed that the 7.5–15 mg dose group had a lower risk of TESAEs than eplerenone (RR = 0.81, 95% CI:0.71–0.92, *P* = 0.002), see Figure 3B. The results of subgroup analysis showed that only the 2.5–5 mg (*P* > 0.68) and 15–20 mg (*P* = 0.07) dose groups of finerenone had a lower risk of TESAEs than eplerenone but had no statistical significance, and there were significant differences in other dose groups in Figure 3B.

### The discontinuation of treatment due to the adverse events in patients with HF

**Figure 4:**
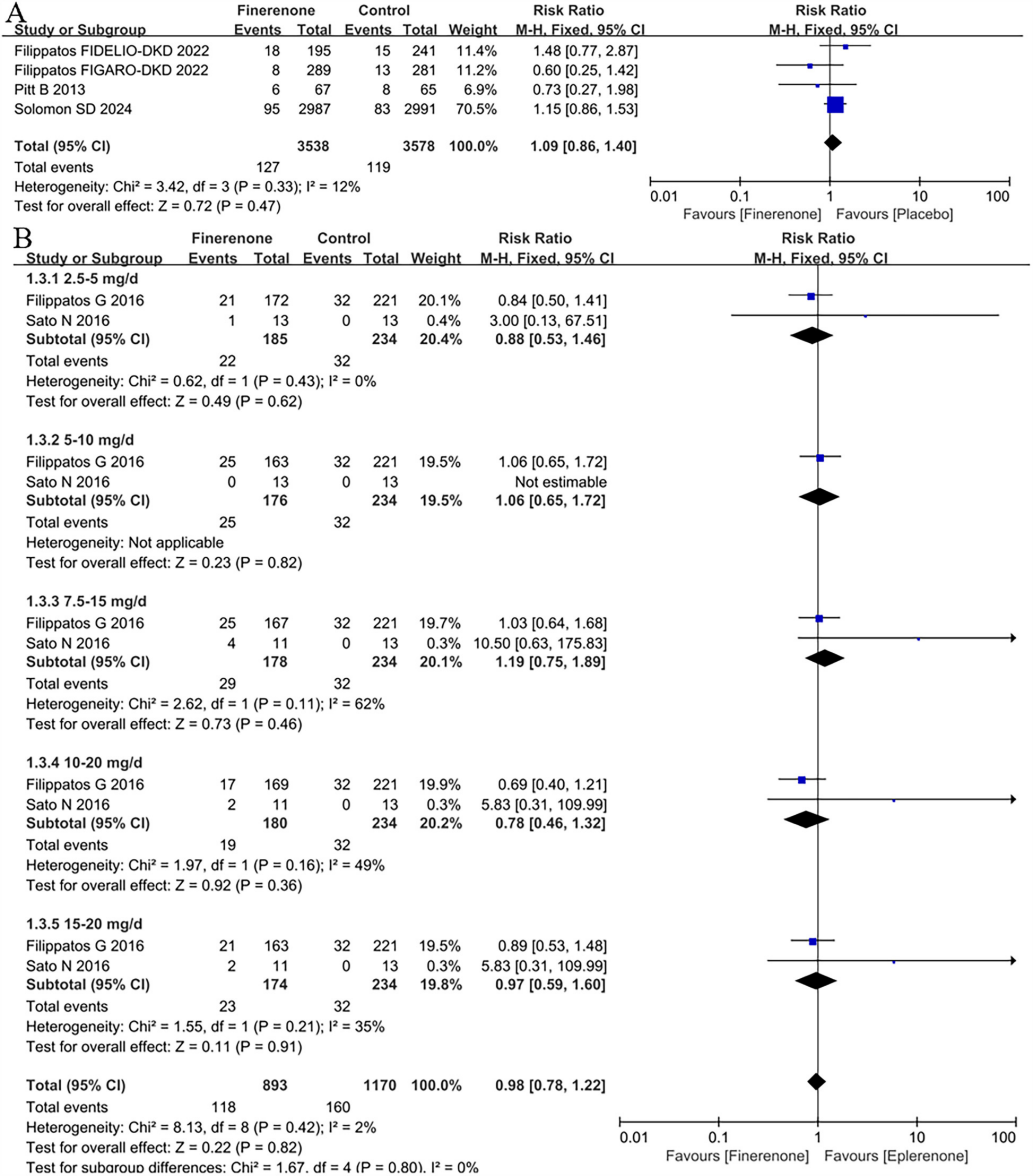
Forest plot of the outcomes of the discontinuation of treatment due to the adverse events. **(A)** the discontinuation of treatment due to the adverse events in patients with HF. **(B)** the discontinuation of treatment due to the adverse events in patients with HFrEF with different dose finerenone. HF, heart failure; HFrEF, heart failure with reduced ejection fraction; HFmrEF, heart failure with mildly reduced ejection fraction; HFpEF, heart failure with preserved ejection fraction.

The RR of the discontinuation of treatment due to the adverse events based on 4 studies (5, 8, 9, 13) was 1.09 (95% CI: 0.86 −1.40, *P* = 0.47; *P* = 0.33 for heterogeneity, *I*^2^ = 12%) in Figure 4A. Although the finerenone group had a 9% higher risk of discontinuing treatment due to adverse events in patients with heart failure, the difference was not statistically significant (*P* = 0.47).

### The discontinuation of treatment due to the adverse events in patients with HFrEF with different dose finerenone

For the discontinuation of treatment due to the adverse events in patients with HFrEF with different dose finerenone, 2 RCTs (11, 12) were included to analysis. There was no difference between finerenone and eplerenone in all dose groups (RR = 0.98, 95% CI 0.78–1.22, *P* = 0.82), see Figure 4B.

### Hyperkalaemia in patients with HF

**Figure 5:**
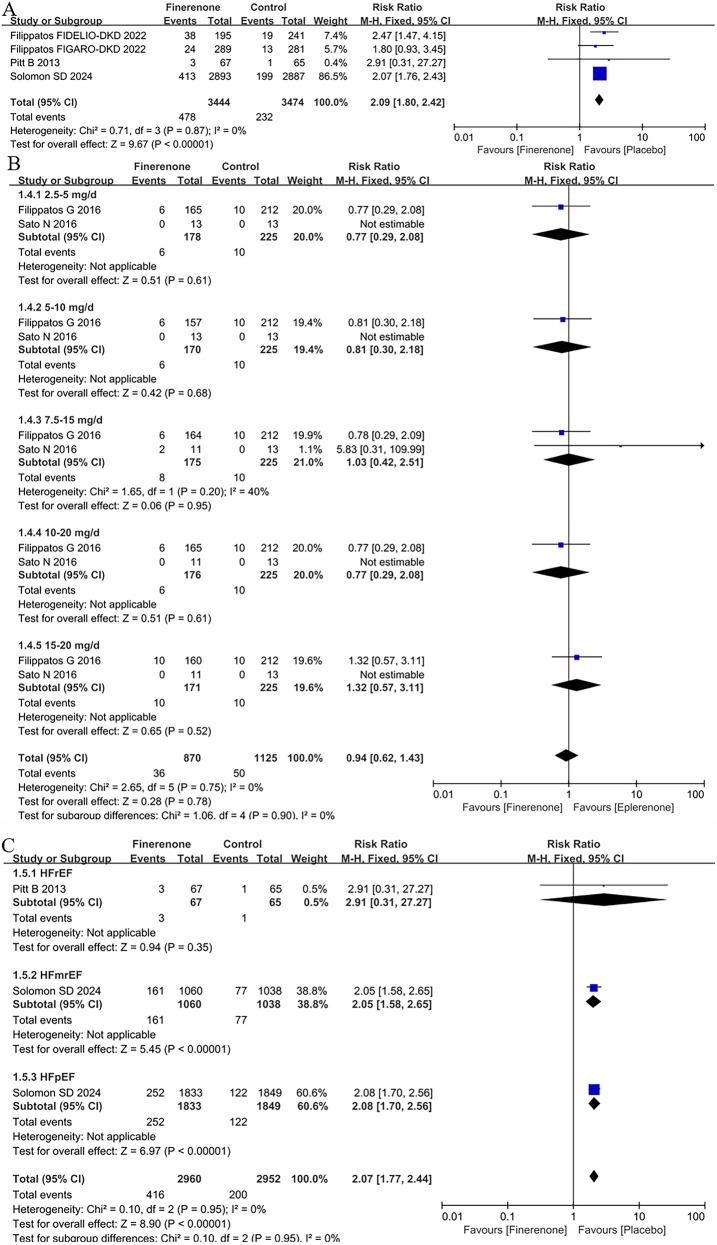
Forest plot of the outcomes of hyperkalaemia. **(A)** Hyperkalaemia in patients with HF. **(B)** Hyperkalaemia in patients with HFrEF with different dose finerenone. **(C)** Hyperkalaemia grouped by heart failure phenotype. HF, heart failure; HFrEF, heart failure with reduced ejection fraction; HFmrEF, heart failure with mildly reduced ejection fraction; HFpEF, heart failure with preserved ejection fraction.

Four trials (5, 8, 9, 13) included in the meta-analysis reported hyperkalaemia. The results showed that patients receiving finerenone had a higher risk of hyperkalaemia than placebo, with a pooled RR of 2.09 (CI: 1.80–2.42, *P* < 0.00001; *P* = 0.087 for heterogeneity, I^2^ = 0%; Figure 5A).

### Hyperkalaemia in patients with HFrEF with different dose finerenone

2 trials (11, 12) included in the meta-analysis reported hyperkalaemia in patients with HFrEF with different dose finerenone. There were no differences for hyperkalaemia in all dose groups [eplerenone 4.4% vs. finerenone 4.1%; RR = 0.94 (95% CI 0.62–1.43), *P* = 0.78] in Figure 5B.

### Hyperkalaemia grouped by heart failure phenotype

2 RCTs (5, 13) provided the data about hyperkalaemia in different HF subtypes. Treatment with finerenone was associated with an increased risk of hyperkalaemia with a prevalence of 6.8% and 14.0% in the placebo and treatment group, respectively [RR = 2.07 (95% CI 1.77–2.44), *P* < 0.00001]. According to HF subpopulation, whereas there was no difference between placebo and finerenone in HFrEF population (RR = 2.91, 95% CI 0.31–27.27, *P* = 0.35), there were a significant higher prevalence of hyperkalaemia in patients treated with finerenone compare to placebo in both HFmrEF (RR = 2.05, 95% CI 1.58–2.65, *P* < 0.00001) and HFpEF [RR = 2.08 (95% CI 1.70–2.65), *P* < 0.00001] in Figure 5C.

### Hypotension grouped by heart failure phenotype

**Figure 6:**
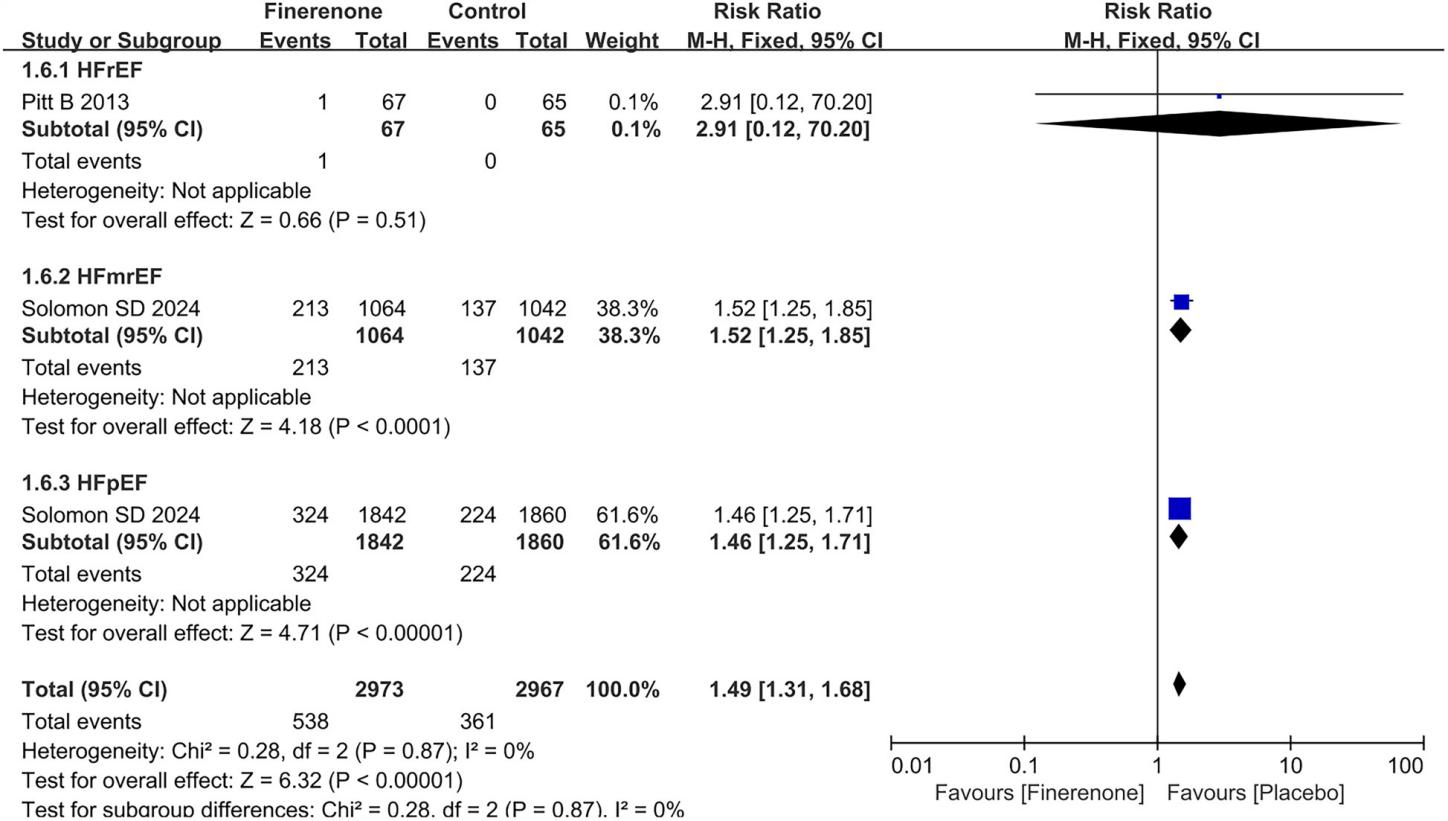
Forest plot of the outcome of hypotension grouped by heart failure phenotype. HF, heart failure; HFrEF, heart failure with reduced ejection fraction; HFmrEF, heart failure with mildly reduced ejection fraction; HFpEF, heart failure with preserved ejection fraction.

Only 2 RCTs (5, 13) reported the result about hypotension in different HF subtypes. Treatment with finerenone was associated with an increased risk of hypotension with a prevalence of 12.2% and 18.1% in the placebo and treatment group, respectively (RR = 1.49, 95% CI 1.31–1.68), *P* < 0.00001). The risk of hypotension was consistency higher in patients treated with finerenone in both HFmrEF (placebo 13.1% vs. finerenone 20.0%; RR = 1.52, 95% CI 1.25–1.85, *P* < 0.0001) and HFpEF [finerenone 17.6% vs. placebo 12.0%; RR = 1.46 (95% CI 1.25–1.71), *P* < 0.00001] population. Conversely in HFrEF, the risk was not statistically increased (placebo 0% vs. finerenone 1.5%; RR = 2.91, 95% CI 0.12–70.20, *P* = 0.51) in Figure 6.

### Comparison of adverse events between different dose finerenone and spironolactone patients with HFrEF

Several adverse events of interest were also compared between different dose finerenone and spironolactone based on one study (5). Although the number of available trials was limited, the results suggested that finerenone may have a lower risk of TEAEs (RR = 0.64, 95% CI 0.56–0.74, *P* < 0.00001), and treatment discontinuation (RR = 0.37, 95% CI 0.25–0.54, *P* < 0.00001) and hyperkalemia (RR = 0.41, 95% CI 0.21–0.79, *P* = 0.008). While individual dose subgroups for hyperkalemia did not reach statistical significance, a significant difference emerged in the pooled analysis. Rates of hypotension were similar between groups (RR = 0.61, 95% CI 0.29–1.30, *P* = 0.20). A detailed summary is provided in Table 3.

**Table 3:** Adverse events of interest compare different dose finerenone to spironolactone in patients with HFrEF.

Adverse events	Intervention vs. comparator	Using fixed-effect models		
		RR, 95%CI	*I* ^2^	*P*
TEAEs	Finerenone vs. spironolactone (25–50 mg q.d) (5)	0.64 [0.56, 0.74]	0	<0.00001
	Finerenone 2.5 mg q.d	0.59 [0.44, 0.79]	0	0.0003
	Finerenone 5.0 mg q.d	0.68 [0.52, 0.87]	0	0.0003
	Finerenone 10 mg q.d	0.64 [0.49, 0.84]	0	0.0001
	Finerenone 5.0 mg bid	0.67 [0.51, 0.87]	0	0.0003
The discontinuation of treatment due to the adverse events	Finerenone vs. spironolactone (25–50 mg q.d) (5)	0.37 [0.25, 0.54]	0	<0.00001
	Finerenone 2.5 mg q.d	0.45 [0.22, 0.92]	0	0.03
	Finerenone 5.0 mg q.d	0.20 [0.07, 0.55]	0	0.002
	Finerenone 10 mg q.d	0.30 [0.13, 0.70]	0	0.005
	Finerenone 5.0 mg bid	0.52 [0.26, 1.02]	0	0.06
Hypotension	Finerenone vs. spironolactone (25–50 mg q.d) (5)	0.61 [0.29, 1.30]	0	0.20
	Finerenone 2.5 mg q.d	0.11 [0.01, 1.93]	0	0.13
	Finerenone 5.0 mg q.d	0.47 [0.09, 2.48]	0	0.37
	Finerenone 10 mg q.d	0.24 [0.03, 2.05]	0	0.19
	Finerenone 5.0 mg bid	1.72 [0.53, 5.60]	0	0.37
Hyperkalemia	Finerenone vs. spironolactone (25–50 mg q.d) (5)	0.41 [0.21, 0.79]	0	0.008
	Finerenone 2.5 mg q.d	0.41 [0.11, 1.51]	0	0.18
	Finerenone 5.0 mg q.d	0.13 [0.02, 1.06]	0	0.06
	Finerenone 10 mg q.d	0.40 [0.11, 1.49]	0	0.17
	Finerenone 5.0 mg bid	0.70 [0.24, 2.10]	0	0.53

HFrEF, heart failure with reduced ejection fraction; TEAEs, treatment-emergent adverse events; RR, risk ratio.

### Risk of bias and sensitivity analysis

A funnel plot is drawn using TEAEs, TESAEs, hyperkalemia, and the discontinuation of treatment due to the adverse events as indicators, see Supplementary Figure S2. It can be seen from the figure that the scattered points of the study are distributed within the funnel plot and have good symmetry, indicating that there is little possibility of publication bias in this study.

We conducted sensitivity analyses by using the fixed-effect model, and sequentially deleting each study and reanalysing the datasets of all remaining studies. Similar results were observed for the primary composite cardiovascular outcome and other outcomes of interest in all conducted sensitivity analyses, revealing high reliability of the result of meta-analysis of outcomes of interest, see Supplementary Table S2.

## Discussion

### Interpretation of key findings

This systematic review and meta-analysis demonstrated an increased risk of hyperkalemia with finerenone compared to placebo, irrespective of HF phenotype (RR = 2.07, 95% CI: 1.77–2.44, *P* < 0.00001). Notably, subgroup analyses showed no significant difference in hyperkalemia risk between finerenone and placebo specifically in patients with HFrEF (RR = 2.91, 95% CI: 0.31–27.27, *P* = 0.35). although the overall risk was elevated, this finding appears to be largely influenced by the results in HFmrEF and HFpEF subgroups. Given that the FINEARTS-HF trial (13)—a large placebo-controlled study—contributed the majority of the analytical weight, interpretation of pooled results should consider this study's dominant impact on the overall estimate.

Additionally, finerenone increased the risk of hypotension compared to placebo across all HF phenotypes (RR = 1.49, 95% CI: 1.31–1.68, *P* < 0.00001). Subgroup analysis indicated a higher risk specifically among patients with HFmrEF (RR = 1.52, 95% CI: 1.25–1.85, *P* < 0.0001) and HFpEF (RR = 1.46, 95% CI: 1.25–1.71, *P* < 0.00001), while no significant difference was observed for patients with HFrEF (RR = 2.91, 95% CI: 0.12–70.20, *P* = 0.51).

When comparing finerenone to eplerenone, no significant differences in the risk of hyperkalemia were observed across various finerenone doses (ranging from 2.0 mg to 20 mg). This may indicate a true similarity in risk profiles or, alternatively, reflect limited statistical power due to the smaller sample sizes of the contributing studies. Notably, this comparison should be interpreted with caution, as the trials involving eplerenone contributed substantially less weight to the meta-analysis compared to the FINEARTS-HF trial (13), which used higher finerenone doses (25 mg or 40 mg).

In addition to comparisons with placebo and eplerenone, several adverse events of interest were also evaluated between different doses of finerenone and spironolactone based on one included study in the HFrEF population. Although limited by the number of trials, the results indicated that finerenone may be associated with a significantly lower risk of TEAEs (RR = 0.64, 95% CI: 0.56–0.74, *P* < 0.00001), treatment discontinuation due to adverse events (RR = 0.37, 95% CI: 0.25–0.54, *P* < 0.00001), and hyperkalemia (RR = 0.41, 95% CI: 0.21–0.79, *P* = 0.008). Although subgroup analyses of individual dose levels for hyperkalemia did not reach statistical significance, the pooled analysis revealed a consistent benefit. No significant difference in the incidence of hypotension was observed between finerenone and spironolactone (RR = 0.61, 95% CI: 0.29–1.30, *P* = 0.20), suggesting comparable blood pressure safety profiles. Interestingly, the discrepancy between the nonsignificant findings in dose-specific subgroups and the significant result in the pooled analysis is likely due to limited sample sizes within each subgroup, reducing the statistical power to detect differences. The aggregated analysis, with its larger cumulative sample size, provided greater power and revealed a significant reduction in hyperkalemia risk with finerenone. These findings imply that while finerenone and spironolactone share similar mechanisms of action as mineralocorticoid receptor antagonists (MRAs), finerenone may offer a more favorable electrolyte safety profile in patients with HFrEF, although this hypothesis requires further validation through larger RCTs.

### Clinical implications and safety profile

Finerenone is a non-steroidal MRA with a higher selectivity and lipophilicity compared to traditional steroidal MRAs such as eplerenone. It distributes evenly in the heart and kidney, efficiently blocks mineralocorticoid receptor (MR) activity at lower doses, and has less interference with glucocorticoid and androgen receptors, potentially reducing adverse effects like gynecomastia or sexual dysfunction (14–16).

Our findings indicate a significantly higher risk of hyperkalemia with finerenone vs. placebo, regardless of HF phenotype. However, there was no dose-dependent difference in the risk of hyperkalemia compared with eplerenone, which may be attributed to its highly selective inhibitory effect, which stabilizes the risk of hyperkalemia (7).The reason for this phenomenon may be that finerenone, its structure, is highly specific for the regulation of MR activity and inhibits sodium reabsorption and enhances potassium retention by blocking the effect of MR in the distal convoluted tubule and collecting duct, resulting in increased risk of hyperkalemia (6). Nonetheless, these outcomes primarily reflect data from the placebo-controlled FINEARTS-HF trial (13), underscoring the need for careful interpretation of dose-related outcomes from smaller studies.

According to our study, finerenone significantly increased the risk of hypotension in patients with HFmrEF and HFpEF, while no difference was seen in patients with HFrEF. Differences in HF phenotypes may be due to variations in hemodynamic status and MR-mediated vascular effects among subtypes (17). Patients with HFmrEF and HFpEF often demonstrate higher vascular resistance and a greater reliance on blood pressure for adequate organ perfusion, whereas those with HFrEF typically have impaired systolic function and lower peripheral resistance, potentially attenuating the hemodynamic impact of vasodilatory therapies (18).

From a mechanistic perspective, the vasodilatory effects of finerenone may arise from its ability to regulate vascular smooth muscle tone via inhibition of the renin–angiotensin–aldosterone system (RAAS) and potential interference with L-type calcium channels, thereby reducing vascular contractility (18). These effects are further supported by its anti-inflammatory and antioxidant properties (19–21), which enhance endothelial function and vascular compliance, contributing to modest blood pressure reductions, particularly in HFmrEF and HFpEF.

Despite this, the antihypertensive effect of finerenone is relatively mild compared to traditional agents (22). Its main therapeutic impact lies in RAAS modulation, addressing neurohormonal dysregulation in HF. This distinct pharmacodynamic profile may underlie the differential incidence of hypotension observed across HF phenotypes.

Importantly, in patients with HFrEF, the absence of significant hypotension risk is consistent with findings from recent guideline (23). According to the 2025 ESC Heart Failure Association consensus (23), asymptomatic or mildly symptomatic low blood pressure—particularly with SBP ≥80 mmHg—should not be a reason to reduce or discontinue guideline-directed medical therapy (GDMT), including finerenone. Instead, emphasis should be placed on individualized clinical assessment. In cases of symptomatic hypotension, particularly in HFmrEF or HFpEF, clinicians should first investigate reversible factors such as over-diuresis, concurrent antihypertensives, or volume depletion. Dose titration strategies should prioritize medications with minimal blood pressure effects, such as SGLT2 inhibitors and MRAs, and employ careful sequencing of beta-blockers or ARNi. Agents such as ivabradine may support heart rate control without additional hypotensive risk. In some cases, decongestion with reduced diuretic dosing and avoidance of unnecessary antihypertensives may help preserve therapeutic dosing of finerenone. Furthermore, as emphasized in the same consensus (23), the management of finerenone-associated hyperkalemia should adhere to guideline-recommended strategies and be guided by individualized risk–benefit assessments. The presence of asymptomatic or mildly symptomatic hyperkalemia alone should not prompt automatic discontinuation of RAAS inhibitors or MRAs. Instead, a stepwise and multidisciplinary approach is advised to safely optimize GDMT while minimizing adverse events.

In this context, our data also demonstrated a favorable safety profile for finerenone. Compared with placebo, it did not significantly increase the risk of TEAEs (RR = 0.95, 95% CI = 0.90–1.01, *P* = 0.09) or TESAEs (RR = 0.99, 95% CI 0.91–1.07, *P* = 0.74). Notably, the risk of TEAEs was significantly lower in the finerenone group compared to eplerenone (RR = 0.93, 95% CI: 0.89–0.98, *P* = 0.008), with the 7.5–15 mg/day subgroup showing a particularly favorable profile (RR = 0.81, 95% CI: 0.71–0.92, *P* = 0.002.). Meanwhile, the 2.5–5 mg (RR = 0.95, 95% CI: 0.75–1.21, *P* = 0.68) and 15–20 mg (RR = 0.79, 95% CI: 0.61–1.02, *P* = 0.07) subgroups showed no significant difference compared with eplerenone, suggesting potential dose-dependent effects. These findings align with prior evidence suggesting that mid-range dosing may offer optimal efficacy and tolerability (24, 25). No significant differences were observed in treatment discontinuation across different doses, and hypotension events were rarely severe enough to warrant withdrawal (26). This supports the notion that finerenone, when properly titrated and monitored, can be safely implemented in clinical practice, including in patients at risk of low blood pressure. This is consistent with previous research showing that patients treated with finerenone have a lower overall risk of serious adverse events, especially among those with a history of heart failure (6, 27).

### Limitations

Our study has several limitations. First, our conclusions primarily derive from the large FINEARTS-HF trial, which alone randomized 6,001 patients with HFmrEF/HFpEF. This trial's dominance significantly influences the overall results and limits their generalizability to other heart failure populations. Second, due to the limited number of reported outcome events, missing data, and variability in subgroup sizes—such as only 132 patients in the HFrEF hypotension subgroup compared to larger groups for HFmrEF and HFpEF—the comprehensiveness of subgroup analyses was restricted. Third, we excluded unpublished studies and non-English language articles, potentially introducing selection bias. Fourth, only one or two included studies directly compared finerenone with steroidal MRAs (spironolactone and eplerenone), and limited high-quality RCTs restrict robust comparisons and validations. Fifth, the trials included in our meta-analysis predominantly enrolled patients with chronic kidney disease rather than overt heart failure, limiting applicability to broader heart failure populations and raising concerns about clinical heterogeneity despite the absence of significant statistical heterogeneity. Lastly, variability in finerenone dosing and other confounding factors across the included RCTs may have introduced additional bias.

### Future directions

This study provides important safety data for the use of finerenone in different heart failure phenotypes. Although it increases the risk of hyperkalemia and hypotension, the overall safety profile is comparable to eplerenone or placebo, which provides theoretical support for its use in the treatment of HF. Future research should focus on:

(1) Further study on the use of finerenone in patients with HF. (2) To explore the optimal dose and use schedule of finerenone in patients with different HF phenotypes; (3) In-depth analysis of the mechanisms of hyperkalemia and hypotension and development of targeted risk control strategies; (4) To evaluate the impact of long-term use of finerenone on patient outcomes; (5) Conducting dedicated trials to directly compare finerenone with other steroidal MRAs (e.g., eplerenone or spironolactone), particularly in populations with HFrEF.

## Conclusion

In conclusion, finerenone (10–25 mg), as primarily evaluated in the FINEARTS-HF trial, demonstrated a safety profile comparable to that of placebo in the overall heart failure population, with no significant differences in TEAEs, TESAEs, or treatment discontinuation. Compared to eplerenone, it was associated with fewer adverse events, and may also present a more favorable safety profile than spironolactone in patients with HFrEF. These findings support the potential role of finerenone as a well-tolerated therapeutic option in heart failure, although further confirmation in diverse HF populations is warranted.

## Data Availability

The original contributions presented in the study are included in the article/Supplementary Material, further inquiries can be directed to the corresponding authors.
