# Correction: Adverse effects of finerenone in patients with heart failure: a systematic review and meta-analysis

**DOI:** 10.3389/fcvm.2025.1652440

**Published:** 2025-07-25

**Authors:** Wanqian Yu, Fan Luo, Jingan Rao, Guangtao Lei, Qinghua Wu, Wen Shen, Pingping Yang, Ping Li

**Affiliations:** ^1^ Department of Cardiovascular Medicine, The Second Affiliated Hospital, Jiangxi Medical College, Nanchang University, Nanchang, Jiangxi, China; ^2^ Department of Gastroenterology, Jiangxi Provincial Hospital of Traditional Chinese Medicine, Nanchang, Jiangxi, China; ^3^ Department of Endocrinology and Metabolism, The Second Affiliated Hospital of Nanchang University, Nanchang, Jiangxi, China

**Keywords:** finerenone, heart failure, HFrEF, HFmrEF, HFpEF, adverse effects

In the abstract, The risk estimate for hyperkalemia was reported incorrectly as (RR = 2.07, 95% CI 1.77–2.44). This has been corrected to read:

Finerenone significantly increased the risk of hyperkalemia (RR = 2.09, 95% CI 1.80–2.42, *P* < 0.00001).

The original version of this article has been updated.

The risk estimate in the 7.5–15 mg dose group was incorrectly reported as RR = 0.81, 95% CI:0.71–0.92, *P* = 0.002. The *p*-value for the 2.5–5 mg dose group was incorrectly reported as *P* > 0.68.

A correction has been made to the section Results, subsection TESAEs in patients with HFrEF with different dose finerenone:

“2 RCTs (11–12) were included the analysis of TESAEs to compare different dose finerenone with eplerenone. The results showed that finerenone had a lower risk of TESAEs than eplerenone (RR = 0.74,95% CI:0.66–0.84, *P* < 0.00001); subgroup analysis showed that the 7.5–15 mg dose group had a lower risk of TESAEs than eplerenone (RR = 0.70, 95% CI: 0.54–0.93, *P* = 0.01), see Fig. 3B. The results of subgroup analysis showed that only the 2.5–5 mg (*P* = 0.68) and 15–20 mg (*P* = 0.07) dose groups of finerenone had a lower risk of TESAEs than eplerenone but had no statistical significance, and there were significant differences in other dose groups in Fig. 3B.”

The original version of this article has been updated.

The heterogeneity *p*-value for hyperkalaemia analysis was incorrectly reported as *P* = 0.087.

A correction has been made to the section Results, subsection Hyperkalaemia in patients with HF:

“Four trials (5, 8–9, 13) included in the meta-analysis reported hyperkalaemia. The results showed that patients receiving finerenone had a higher risk of hyperkalaemia than placebo, with a pooled RR of 2.09 (CI: 1.80–2.42, *P* < 0.00001; *P* = 0.87 for heterogeneity, *I*^2^ = 0%; Figure 5A).”

The original version of this article has been updated.

The confidence interval for the HFpEF subgroup was incorrectly reported as 95% CI 1.70–2.65.

A correction has been made to the section Results, subsection Hyperkalaemia grouped by heart failure phenotype:

“2 RCTs (5, 13) provided the data about hyperkalaemia in different HF subtypes. Treatment with finerenone was associated with an increased risk of hyperkalaemia with a prevalence of 6.8% and 14.0% in the placebo and treatment group, respectively [RR = 2.07 (95% CI 1.77–2.44), *P* < 0.00001]. According to HF subpopulation, whereas there was no difference between placebo and finerenone in HFrEF population (RR = 2.91, 95% CI 0.31–27.27, *P* = 0.35), there were a significant higher prevalence of hyperkalaemia in patients treated with finerenone compare to placebo in both HFmrEF (RR = 2.05, 95% CI 1.58–2.65, *P* < 0.00001) and HFpEF [RR = 2.08 (95% CI 1.70–2.56), *P* < 0.00001] in Figure 5C).”

The original version of this article has been updated.

The risk estimate in the 7.5–15 mg dose group was correctly reported as RR = 0.81, 95% CI:0.71–0.92, *P* = 0.002.

A correction has been made to the section Discussion, subsection Clinical Implications and Safety Profile:

“In this context, our data also demonstrated a favorable safety profile for finerenone. Compared with placebo, it did not significantly increase the risk of TEAEs (RR = 0.95, 95% CI = 0.90–1.01, *P* = 0.09) or TESAEs (RR = 0.99, 95% CI 0.91–1.07, *P* = 0.74). Notably, the risk of TEAEs was significantly lower in the finerenone group compared to eplerenone (RR = 0.93, 95% CI: 0.89–0.98, *P* = 0.008), with the 7.5–15 mg/day subgroup showing a particularly favorable profile (RR = 0.81, 95% CI:0.71–0.92, *P* = 0.002.). Meanwhile, the 2.5–5 mg (RR = 0.95, 95% CI: 0.75–1.21, *P* = 0.68) and 15–20 mg (RR = 0.79, 95% CI: 0.61–1.02, *P* = 0.07) subgroups showed no significant difference compared with eplerenone, suggesting potential dose-dependent effects. These findings align with prior evidence suggesting that mid-range dosing may offer optimal efficacy and tolerability (25–26). No significant differences were observed in treatment discontinuation across different doses, and hypotension events were rarely severe enough to warrant withdrawal (27). This supports the notion that finerenone, when properly titrated and monitored, can be safely implemented in clinical practice, including in patients at risk of low blood pressure. This is consistent with previous research showing that patients treated with finerenone have a lower overall risk of serious adverse events, especially among those with a history of heart failure (17, 28).”

The original version of this article has been updated.

