# Supplementary material for: Adverse effects of finerenone in patients with heart failure: a systematic review and meta-analysis
Source: Front Cardiovasc Med. 2025 May 27;12:1601552. doi: 10.3389/fcvm.2025.1601552 (PMC12149160; doi:10.3389/fcvm.2025.1601552)
Supplement: Supplementary file 1 [file Datasheet1.zip › Supplementary table S1.docx]

**Supplementary table S1. The setting of RCTs.**

**Supplementary table S1A.** The setting of RCTs (Finerenone vs placebo).

| **Characteristic** | **ARTS**  **Pitt B 2013** | | **FIDELIO-DKD**  **Filippatos 2022** | | **FIGARO-DKD**  **Filippatos 2022** | | **FINEARTS-HF**  **Solomon SD 2024** | | **P value** |
| --- | --- | --- | --- | --- | --- | --- | --- | --- | --- |
|  | **Finerenone**  **(N = 264)** | **Placebo**  **(N = 65)** | **Finerenone**  **(N =195)** | **Placebo**  **(N = 241)** | **Finerenone**  **(N = 290)** | **Placebo**  **(N =281)** | **Finerenone**  **(N = 3003)** | **Placebo**  **(N =2998)** |  |
| **Ages (years)** | 75.5 (9.71) | 72.4 (8.5) | 66.4 (8.8) | 66.2 (8.5) | 64.89 (8.95) | 66.32 (8.75) | 71.9（9.6） | 72（9.7） | P＞0.05 |
| **Males** | 212 | 50 | 120 | 160 | 182 | 168 | 1648 | 1621 | P＞0.05 |
| **eGFR, ml/min/1.73 m2, mean (SD)** | 43.3 (10.77) | 46.9 (8.2) | 42.6 (12.9) | 42.1 (12.5) | 63.97 (21.39) | 62.8 (22) | 61.9 (19.4) | 62.3 (20) | P＞0.05 |
| **systolic blood pressure, mmHg, mean (SD)** | 101 (20.2) | 127 (22.5) | 138 (13.6) | 138 (15.1) | 135.71 (13.58) | 134.97 (13.88) | 129.5 (15.3) | 139.3 (15.3) | P＞0.05 |

**Supplementary table S1B.** The setting of RCTs (Finerenone vs eplerenone).

| **Characteristic** | **ARTS-HF**  **Filippatos G 2016** | | **ARTS-HF Japan**  **Sato N 2016** | | **P value** |
| --- | --- | --- | --- | --- | --- |
|  | **Finerenone**  **(N = 834)** | **Placebo**  **(N = 221)** | **Finerenone**  **(N = 59)** | **Placebo**  **(N =13)** |  |
| **Mean age (years)** | 70.83 | 72.4 | 72.38 | 76.5 |  |
| **Males** | 645 | 170 | 41 | 12 | P＞0.05 |
| **eGFR, ml/min/1.73 m2, mean** | 53.2 | 52 | 41.3 | 47.2 |  |
| **systolic blood pressure, mmHg, mean** | 117.8 | 121 | 112.7 | 110.9 |  |
