# Supplementary material for: Adverse effects of finerenone in patients with heart failure: a systematic review and meta-analysis
Source: Front Cardiovasc Med. 2025 May 27;12:1601552. doi: 10.3389/fcvm.2025.1601552 (PMC12149160; doi:10.3389/fcvm.2025.1601552)
Supplement: Supplementary file 1 [file Datasheet1.zip › Supplementary table S2.docx]

**Supplementary table S2.** Sensitivity analyses.

|  | **Using fixed-effect models** | | **sequentially deleting each study and reanalysing the datasets of all remaining studies** | | |
| --- | --- | --- | --- | --- | --- |
|  | RR, 95%CI | I^2^ | Excluded trial | RR, 95%CI | I^2^ |
| **TEAEs** | 0.95 [0.90, 1.01] | 0 | Filippatos FIDELIO-DKD 2022  Filippatos FIGARO-DKD 2022  Pitt B 2013 | 0.94[0.87, 1.01]  0.98[0.89, 1.07]  0.95[0.90, 1.00] | **0**  **0**  **0** |
| **TESAEs** | 0.99 [0.91, 1.07] | 45 | Filippatos FIDELIO-DKD 2022  Filippatos FIGARO-DKD 2022  Solomon SD 2024 | 1.01[0.92, 1.10]  0.97[0.89, 1.06]  0.94[0.79, 1.12] | **0**  **64**  **70** |
| **The discontinuation of treatment due to the adverse events** | 1.09 [0.86, 1.40] | 12 | Filippatos FIDELIO-DKD 2022  Filippatos FIGARO-DKD 2022  Pitt B 2013  Solomon SD 2024 | 1.04[0.80, 1.36]  1.16[0.90, 1.49]  1.12[0.87, 1.44]  0.97[0.62, 1.53] | **20**  **0**  **27**  **36** |
| **Hyperkalaemia** | 2.09 [1.80, 2.42] | 0 | Filippatos FIDELIO-DKD 2022  Filippatos FIGARO-DKD 2022  Pitt B 2013  Solomon SD 2024 | 2.06[1.76, 2.41]  2.11[1.81, 2.46]  2.08[1.80, 2.42]  2.20[1.48, 3.28] | **0**  **0**  **0**  **0** |
