# Supplementary material for: Adverse effects of finerenone in patients with heart failure: a systematic review and meta-analysis
Source: Front Cardiovasc Med. 2025 May 27;12:1601552. doi: 10.3389/fcvm.2025.1601552 (PMC12149160; doi:10.3389/fcvm.2025.1601552)
Supplement: Supplementary file 1 [file Datasheet1.zip › Supplementary figure S1.pptx]

## Slide 1
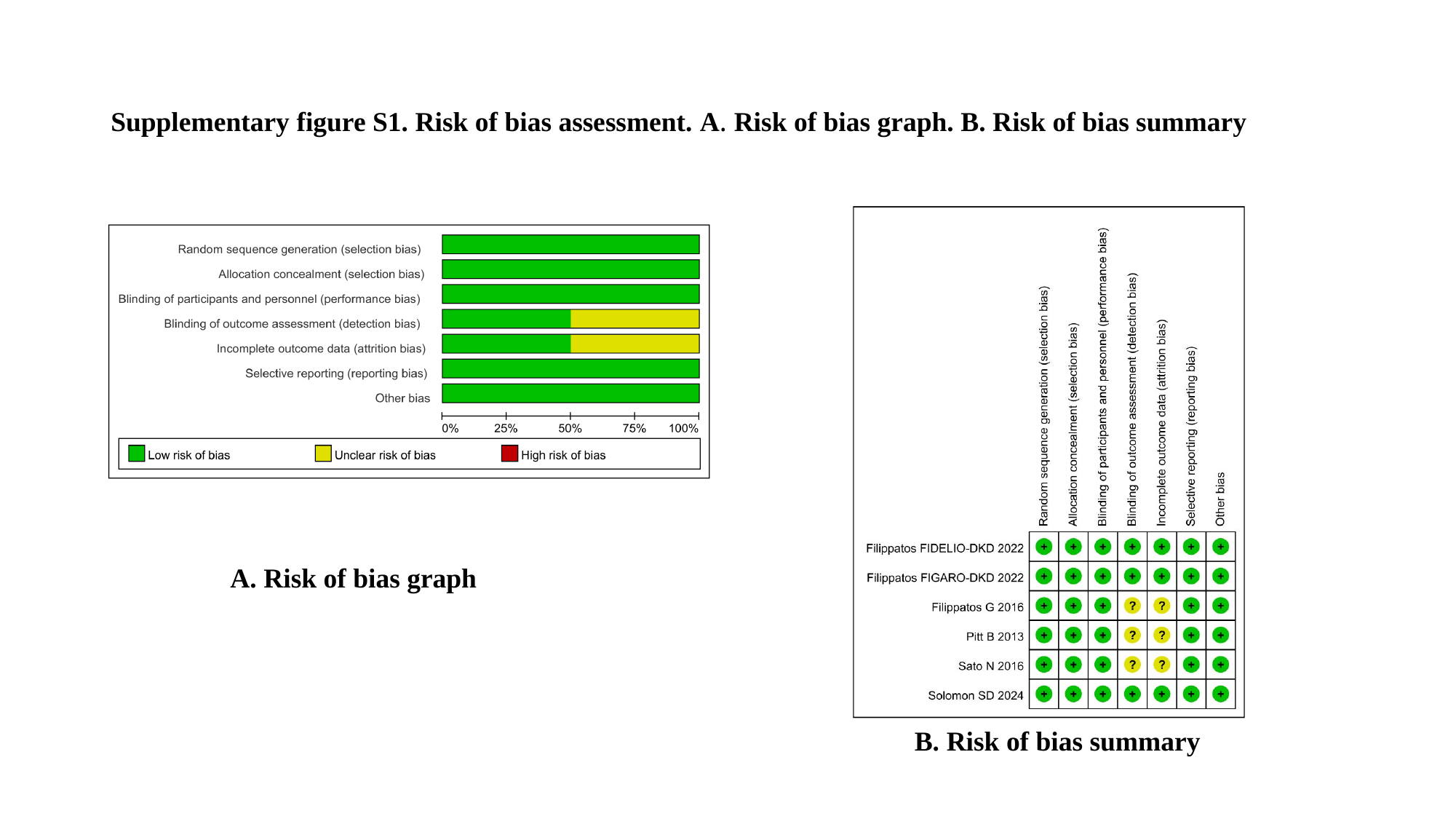

# Supplementary figure S1. Risk of bias assessment. A. Risk of bias graph. B. Risk of bias summary
 A. Risk of bias graph
B. Risk of bias summary
