# Supplementary material for: Adverse effects of finerenone in patients with heart failure: a systematic review and meta-analysis
Source: Front Cardiovasc Med. 2025 May 27;12:1601552. doi: 10.3389/fcvm.2025.1601552 (PMC12149160; doi:10.3389/fcvm.2025.1601552)
Supplement: Supplementary file 1 [file Datasheet1.zip › Supplementary figure S2.pptx]

## Slide 1
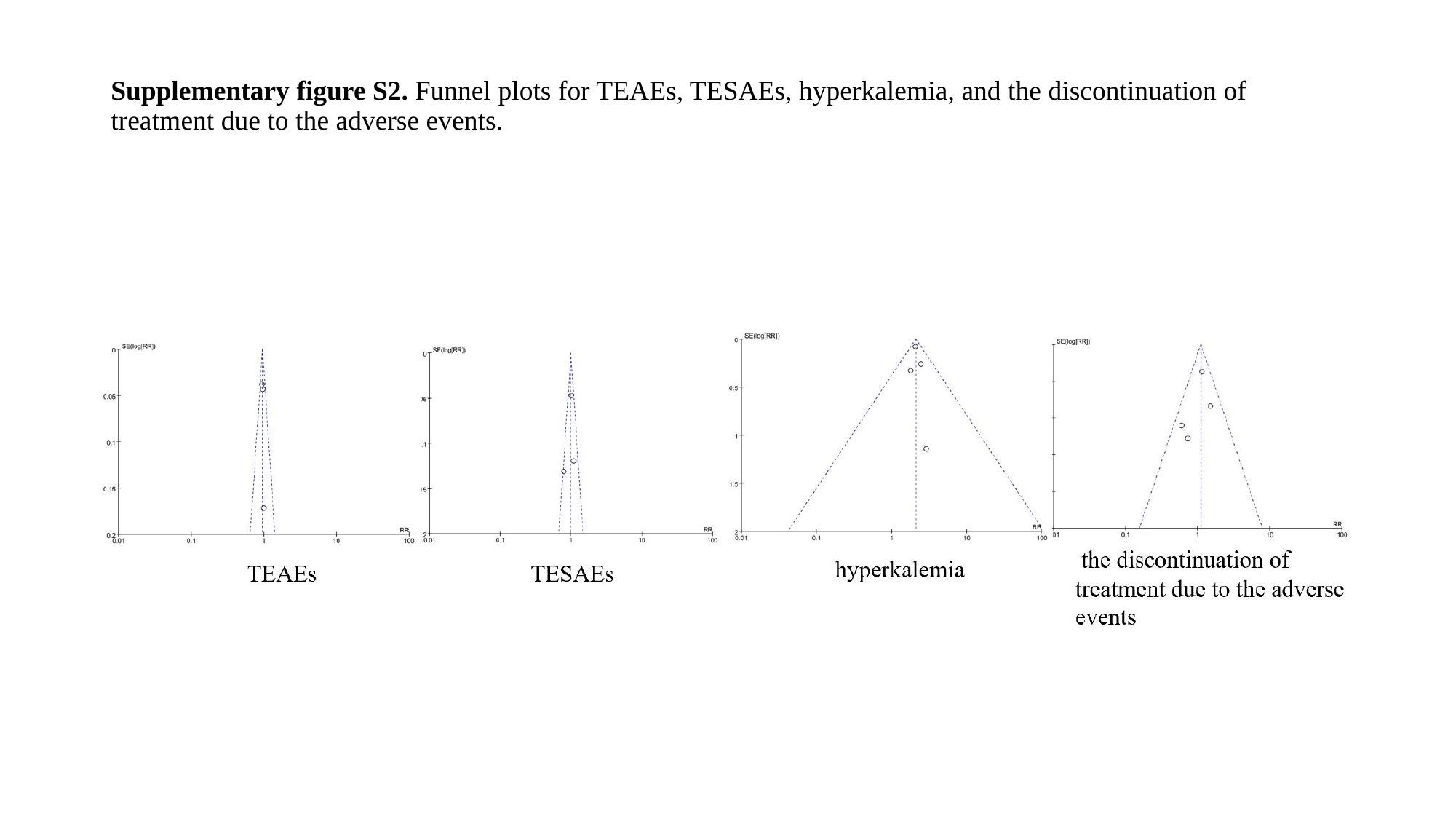

# Supplementary figure S2. Funnel plots for TEAEs, TESAEs, hyperkalemia, and the discontinuation of treatment due to the adverse events.
